# Research Progress on Displacement Mechanism of Supercritical CO_2_ in Low-Permeability Heavy Oil Reservoir and Improvement Mechanism of Displacement Agents

**DOI:** 10.3390/molecules28166154

**Published:** 2023-08-21

**Authors:** Yuanxiu Sun, Weijie Zhang, Jinlong Tian, Yanzhao Meng, Liping Zhang

**Affiliations:** 1 College of Petroleum Engineering, Liaoning Petrochemical University, Fushun 113001, China; 2 Baikouquan Oil Production Plant of Petrochina Xinjiang Oilfield Branch, Karamay 834000, China; pb-tianjl@petrochina.com.cn (J.T.); mengyzh@petrochina.com.cn (Y.M.); 3 D&P Technology Research Institute, Petrochina Liaohe Oilfield Company, Panjin 124000, China; zhanglp02@petrochina.com.cn

**Keywords:** SC-CO_2_, heavy oil, swelling, viscosity, extraction, nanoparticles, polymer, surfactants

## Abstract

With the continuous growth of global energy demand and the late stage of conventional oilfield exploitation, the demand for developing and utilizing low-permeability heavy oil reservoirs is becoming increasingly urgent. However, the exploitation of low-permeability heavy oil reservoirs faces many challenges due to their high viscosity, low permeability, and complex geological conditions. To overcome these challenges, researchers have gradually introduced SC-CO_2_ as an oil displacement agent in the exploitation of heavy oil reservoirs. However, the oil displacement mechanism of SC-CO_2_ in low-permeability heavy oil reservoirs and its improvement mechanism are still not completely understood. The article provides a detailed study and understanding of the oil displacement mechanism of SC-CO_2_, which involves the expansion of heavy oil volume through SC-CO_2_ dissolution. This mechanism reduces the capillary resistance and flow resistance during the oil flow process. The permeation of CO_2_ disrupts the internal structure and arrangement of heavy oil, reducing its viscosity. CO_2_ extracts both light and heavy components from the heavy oil, reducing the residual oil saturation. In addition, the mechanism of improving the effect of oil displacement agents such as nanoparticles, polymers, and surfactants on SC-CO_2_ displacement was also explored. By further exploring the mechanisms and improvement mechanisms of SC-CO_2_ displacement for heavy oil, it can guide the selection and optimization of oil displacement agents. Furthermore, understanding the mechanism can also provide a theoretical basis for engineering practice and technical innovation. While the research on CO_2_ flooding is analyzed and evaluated, the obstacles and challenges that still exist at this stage are indicated, and future research work on CO_2_ in low-permeability heavy oil reservoirs is proposed.

## 1. Introduction

In the 21st century, the increasing global oil and gas demand and the gradual decline in conventional oil and gas production have not met the needs of consumption growth. The effective use of unconventional oil and gas is of key strategic significance in alleviating the contradiction between oil and gas supply and demand, ensuring energy security, promoting a low-carbon transformation of the energy mix, and promoting carbon sequestration [1,2,3]. Chemical flooding has achieved great success in conventional medium-high-permeability reservoirs. However, it has entered a late stage of development after a long period of development. The production gradually decreases, and the reservoir development target gradually shifts from medium-high-permeability reservoirs to low-permeability heavy oil reservoirs.

Improving the recovery efficiency of low-permeability heavy oil reservoirs has become one of the important challenges in the petroleum industry [4]. The main reason is that there is more gum, asphaltene, petroleum wax, and organic acids in heavy oil, which results in crude oil having higher density and viscosity. This leads to insufficient fluidity and makes it unable to be extracted using conventional methods [5]. The other reason is that the pore size of low-permeability reservoirs is small, resulting in a large specific surface area and strong heterogeneity. This makes it difficult for the oil displacement agent to be injected smoothly [6]. The adsorption and retention losses of HPAM, alkali, and surfactant increase, leading to an increase in displacement cost [7,8]. The traditional waterflood mining cannot effectively improve the flow of heavy oil due to its high viscosity. Moreover, there are issues of high initial pressure and high residual oil content in waterflood mining [9]. In addition, the low permeability of reservoirs leads to high displacement pressure, which further increases the difficulty in water flooding [10]. CO_2_ flooding is an oil recovery technology that uses the pressure difference generated after CO_2_ gas injection to drive the crude oil in the formation, making it more fluid and easier to recover. Therefore, CO_2_ flooding has become an effective method to address the poor fluidity of heavy oil and the low injection capacity of oil displacement agents, ultimately improving the oil recovery efficiency of low-permeability heavy oil reservoirs [11]. The field tests show that the oil recovery rate of CO_2_ flooding is 10% higher than that of water flooding. Simultaneously, the reservoir can also partially store CO_2_ to alleviate the greenhouse effect [12]. In the future, CO_2_ flooding technology will link CO_2_ capture and transmission technologies to form a green and sustainable CO_2_ industry chain, providing a two-way drive for environmental protection and oil exploitation.

At present, most of the existing oilfields belong to continental sedimentary reservoirs with high oil viscosity, serious heterogeneity, low water drive recovery, and high recombination content. Most of the newly discovered crude oil is in low-permeability reservoirs, and it is difficult to achieve a high oil recovery rate under conventional production technology. As an oil displacement agent to enhance oil recovery, CO_2_ has attracted extensive attention in scientific research and engineering applications [13,14,15]. Existing studies have shown that CO_2_ in the supercritical state exhibits the characteristics of gas-like viscosity and liquid-like solubility [16]. In most reservoir conditions, CO_2_ exists in a supercritical state, which can reduce the viscosity of crude oil, improve the oil–water mobility ratio, and cause crude oil to expand. At the same time, it can also extract the light hydrocarbon components from crude oil and effectively reduce the interfacial tension between oil and water, thereby increasing the oil washing efficiency and significantly increasing the crude oil recovery [17,18,19,20,21].

An important mechanism of CO_2_ flooding to enhance oil recovery is that CO_2_ can be easily dissolved into crude oil and cause a swelling effect. CO_2_ is more soluble in oil than common hydrocarbon gases, and its solubility in crude oil is greater than its solubility in water, thus supplementing reservoir pressure and increasing oil saturation. The dissolution of CO_2_ expands the volume of crude oil, which can increase the energy of elasticity of the formation and thus increase the internal kinetic energy of crude oil [22]. The viscosity of crude oil will decrease significantly when CO_2_ is dissolved in crude oil, which mainly depends on pressure and temperature of the reservoir, and the viscosity of the non-carbonated crude oil. It effectively expands the swept area of crude oil and improves the mobility ratio of crude oil. The flow of crude oil is improved, thus improving the efficiency of oil displacement [23,24,25]. CO_2_ extracts or vaporizes the light components in crude oil due to which the surface tension at the oil–water interface is reduced, and the remaining oil saturation is also reduced, thus improving oil recovery. CO_2_ can preferentially extract and vaporize light hydrocarbons in crude oil, and then heavier hydrocarbons will also be vaporized. After the hydrocarbon components in crude oil are extracted, the oil–water interfacial tension decreases after continuous enrichment [26,27,28,29]. CO_2_ occupies a certain gap in the oil reservoir, which increases the pressure of the oil reservoir and increases the crude oil production. During the production process, the pressure drop causes the CO_2_ dissolved in the crude oil to escape, which has the effect of dissolved gas flooding and improves the oil displacement effect [30,31].

Due to the limitation of experimental conditions, the relevant studies on the mechanism of swelling and viscosity reduction of heavy oil by SC-CO_2_ are still relatively few, and the theoretical basis is relatively weak, and the microscopic mechanism of fluid interaction is still unclear. In view of the above reasons, the characteristics and microscopic mechanism of SC-CO_2_ displacement in low-permeability heavy oil reservoirs are discussed. The aim is to further understand the mechanism of action of CO_2_ and effectively pave the way for further improvements in oil recovery. Asphaltene deposition is an inevitable problem during CO_2_ flooding in low-permeability reservoirs, which is affected by many factors. Asphalt has a complex structure and contains heteroatoms such as nitrogen, sulfur, or oxygen, making it the most polar and surfactant-like component in crude oil [32]. The main mechanisms by which nanomaterials enhance oil recovery include changes in wettability, reduction in interfacial tension between oil and water, and alteration in oil–water viscosity ratio [33]. The strong polarity of asphaltene can be used to inhibit the deposition of asphaltene and stably disperse asphaltene in the crude oil system under the action of nanoparticles [34,35,36,37]. CO_2_ foams are commonly used in reservoirs where CO_2_ is supercritical. When CO_2_ is supercritical, it forms a weak and unstable foam [38,39,40]. In addition, in low-permeability heavy oil reservoirs, gas channeling and low oil recovery are inevitable under SC-CO_2_ displacement [41,42,43]. HPAM enters high-permeability layers by flowing along large channels and by stretching deformations, thereby reducing the permeability of the high-permeability layers. It improves the heterogeneity of the reservoir, expands the swept volume, and thereby increases the EOR. Most oil reservoirs have excessively high miscibility pressure between crude oil and CO_2_, which prevents successful miscible displacement. During enhanced oil recovery, CO_2_ is utilized to carry surfactants into the formation and act on the oil–gas interface. It reduces the interfacial tension between oil and water, thereby lowering the minimum miscibility pressure of the system and compensating for the drawbacks of existing methods [44,45]. Therefore, this paper studies and discusses the effect and interaction mechanism of oil displacement agents, such as nanoparticles and polymers, and surface activity on CO_2_ flooding, highlighting the improvement technology of CO_2_ flooding. While the research on CO_2_ flooding is analyzed and evaluated, the obstacles and challenges that still exist at this stage are indicated, and future research work on CO_2_ in low-permeability heavy oil reservoirs is proposed.

## 2. Overview of SC-CO_2_ and Heavy Oil

### 2.1. Basic Properties of SC-CO_2_

**Figure 1:**
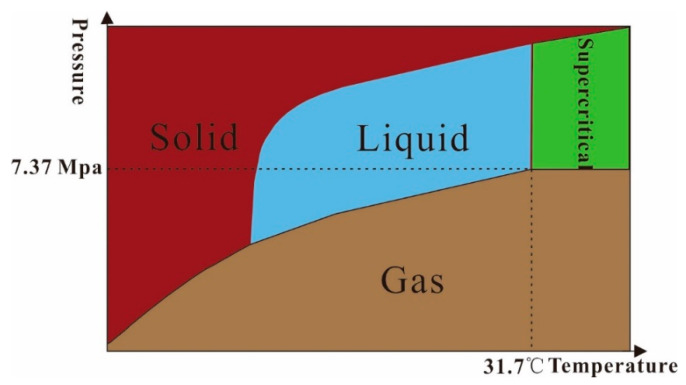
Phase change in CO_2_.

A substance can be present in three states, i.e., solid, liquid, and gas, with the change in ambient pressure and temperature. Any pressure can convert the substance from a gaseous state to a liquid state. When the temperature of a substance is above a specific value, the temperature at that point is called the critical temperature Tc. The lowest pressure at which a gaseous state can be converted to a liquid state is called the critical pressure Pc at the critical temperature. A supercritical fluid (SCF) is a fluid whose temperature and pressure are at the critical temperature (Tc) and critical pressure (Pc), respectively. SC-CO_2_ refers to a fluid in which the temperature and pressure exceed its critical point. The critical temperature (Tc) of CO_2_ is about 31.7 °C, and the critical pressure (Pc) is about 7.37 MPa [46]. The phase diagram of SC-CO_2_ is shown in Figure 1.

The green area in the diagram is the supercritical state. Currently, the most studied supercritical fluid is CO_2_. SC-CO_2_ is used in a wide range of industrial manufacturing applications including electrochemical deposition, preparation of nanoparticles, processing of hard-to-cut materials, cleaning of precision instruments, and centrifugal compression of petroleum [40,47,48,49]. CO_2_ at its critical temperature and pressure has a higher density, and as the temperature increases, the diffusion coefficient of CO_2_ gradually increases while the viscosity decreases. As the pressure increases, the diffusion coefficient of CO_2_ gradually decreases, while the viscosity increases. Under normal conditions, the density of SC-CO_2_ is generally similar to that of crude oil, and the low viscosity and high diffusivity of SC-CO_2_ enable it to rapidly diffuse into the narrow pores of the reservoir, thereby displacing resident oil and gas. In addition, the conditions required for CO_2_ to reach a supercritical state are low, making these conditions less demanding on production equipment and highly operable. As a non-toxic and odorless environmentally friendly gas, CO_2_ can be recovered and reused from industrial waste gases, enabling industrial production and improving economic efficiency. At the same time, it can alleviate environmental problems such as the greenhouse effect faced by the world to a certain extent. Furthermore, CO_2_ exhibits strong solubility for substances with lower molecular weights or weaker polarity, such as alkanes below C_18_.

### 2.2. Composition and Viscous Mechanism of Heavy Oil

Heavy oil is a complex product composed of light components such as saturated hydrocarbons and aromatic hydrocarbons, as well as heavy components such as resins and asphaltenes in a certain proportion. The reason why heavy oil is “thick” is because of its high proportion of gum and asphaltene. There are many studies and assumptions about the structure and adhesion mechanism of gum and asphaltene, and it is generally believed that asphaltene is the main influencing factor.

**Figure 2:**
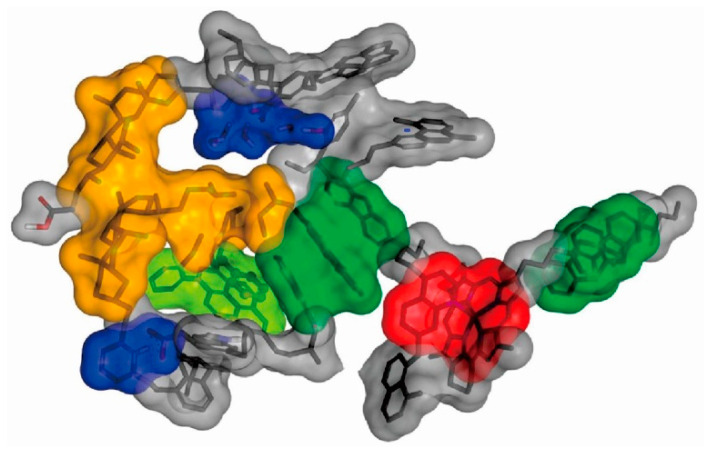
Supramolecular assembly structure of asphaltenes: acid-base interactions and hydrogenbonding (blue), metal coordination complex (red), a hydrophobic pocket (orange), π-π stacking (face-to-face dark green; within a clathrate containing toluene, light green) [52].

The individual molecules of asphaltene exhibit strong polarity and contain more aromatic heterocyclic structures. These aromatic heterocyclic structures are stacked with each other by π-π conjugation, and this stacking structure can be further stabilized by hydrogen bonding of oxygen, nitrogen, sulfur, and other heteroatoms in asphaltene or the chelation of metal heteroatoms such as Ni and V in heavy oil, forming asphaltene supramolecular structures [50,51]. Asphaltenes are encapsulated by colloidal molecules with polar groups that form a surrounding coating, resulting in asphaltene particles encapsulated by colloids that are also highly polar and further leading to their aggregation through hydrogen bonding and eventually forming large molecular aggregates. This phenomenon contributes to the high viscosity of heavy oil. Through hydrogen bonding, they further accumulate and eventually form macromolecular aggregates, resulting in high viscosity of heavy oil. Figure 2 shows a supramolecular assembly structure of asphaltene aggregates proposed by Gray et al. They believe that the asphaltene aggregates are supramolecular structures formed by hydrogen bonding, metal chelation, van der Waals force, π-π interaction, and acid–base interaction [52].

Additionally, heavy oil easily forms stable W/O type emulsions with small amounts of water, resulting in increased viscosity. Processing and treating this stable W/O type emulsion of heavy oil is relatively difficult and challenging in the petroleum industry. The presence of asphaltene is conducive to the stability of W/O emulsions because the asphaltene molecule itself is a natural surfactant. Its polar functional groups such as metal heteroatoms have strong hydrophilicity, while the aromatic ring structure and hydrocarbon chain in the molecule are hydrophobic. The amphiphilicity of asphaltene molecules enables them to adsorb at the oil–water interface, forming an interfacial film with a certain mechanical strength, thus preventing water droplets from gathering and enhancing the stability of W/O emulsions [53].

### 2.3. Geological Characteristics of Low-Permeability Reservoirs and EOR Techniques—CO_2_ Flooding

With the continuous development of onshore oilfields, old oilfields have entered the late stage of high water cut, and the focus of petroleum exploration and development has gradually shifted to low-permeability reservoirs. Low-permeability reservoirs have abundant reserves both domestically and internationally, with recoverable reserves reaching 483 × 10^8^ t [54]. Low-permeability oil and gas reservoirs refer to reservoirs with low porosity, poor fluid flow capacity, and low natural productivity, making it difficult to effectively develop them using conventional extraction methods. Currently, there is no unified classification standard for low-permeability reservoirs both domestically and internationally. In the US, reservoirs with permeability below 10 mD are classified as medium to poor reservoirs. In China, low-permeability reservoirs are divided into low-permeability (10–50 mD), ultra-low-permeability (1–10 mD), and super-low-permeability reservoirs (0.1–1 mD) [55,56,57,58,59]. Low-permeability oil reservoirs are predominantly lithologic reservoirs, characterized by a single type, mainly elastic drive reservoir. Its reservoir physical properties are poor, with low porosity and permeability, small pore throats, and strong heterogeneity. However, it has a high oil saturation and favorable crude oil properties. Therefore, the low-permeability oil reservoir has enormous potential and high exploitation value [60,61,62,63].

The United States is the country with the fastest development and the highest number of projects in CO_2_ flooding technology. In the 121 projects implemented in 2012, 75.8% of the reservoirs were classified as low-permeability oil reservoirs, indicating the significant importance of CO_2_ flooding in EOR for low-permeability and ultra-low-permeability reservoirs [64]. Compared to other gases, CO_2_ has a lower critical temperature and critical pressure, generating a more favorable miscibility with crude oil. Even without miscibility, CO_2_ still exhibits excellent oil displacement effects. After dissolving in crude oil, CO_2_ causes the oil to expand and reduces its viscosity, making it easier to transport. Under certain pressure, CO_2_ can extract both light and heavy components, causing the composition of the crude oil to approach equilibrium. CO_2_ dissolved in crude oil can also reduce interfacial tension. The weak acidity of CO_2_ can play a role in plugging in acidification to some extent.

The injection of external fluids complicates the flow relationship between oil, water, and gas in low-permeability reservoirs. During the oil displacement process, physical and chemical reactions such as convection, diffusion, adsorption, and ion exchange often occur. Studying the oil displacement mechanism of carbon dioxide in low-permeability reservoirs is crucial for injection-production parameters optimization and effect forecasting. Therefore, this study elaborates on the oil displacement mechanism of carbon dioxide in low-permeability heavy oil reservoirs and the improvement mechanism in Section 3 and Section 4. The ecological environment in low-permeability oilfields is fragile, and the contradiction between efficient exploitation of petroleum resources and environmental protection is becoming increasingly prominent. CO_2_ flooding can achieve the storage of greenhouse gases and improve the oil recovery rate, making it a highly promising technology for enhanced oil recovery in low-permeability reservoirs.

## 3. Main Mechanism of SC-CO_2_ Flooding of Heavy Oil

**Figure 3:**
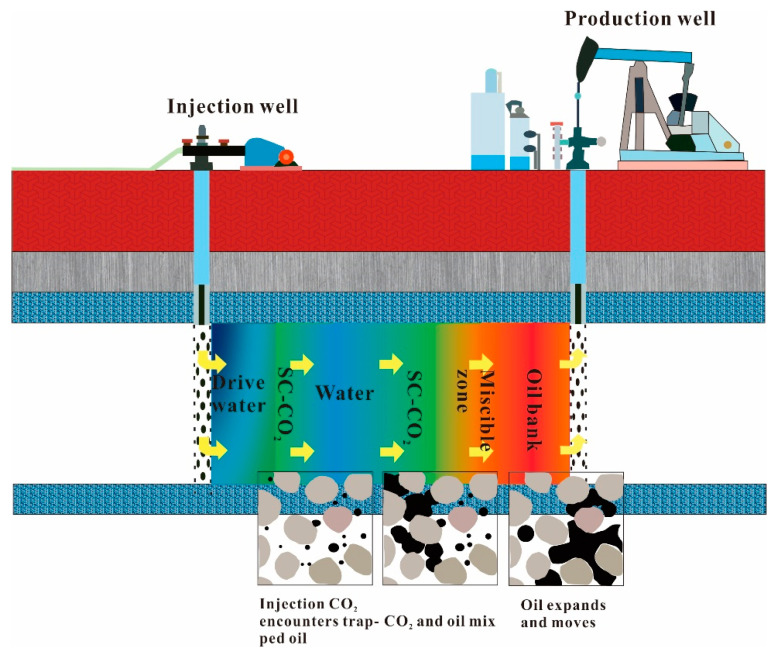
The principle of SC-CO_2_ miscible flooding technology.

CO_2_ flooding is one of the oil displacement methods in tertiary oil recovery, and SC-CO_2_ miscible drive is one of them. After reaching the reservoir, CO_2_ becomes supercritical. It displaces oil from the large pores and mixes with it, forming an oil bank ahead of the miscible zone. The SC-CO_2_ zone is followed by the water lock, which drives oil from the reservoir to the bottom of the production well, ultimately allowing oil to be recovered. The working principle diagram of SC-CO_2_ miscible flooding technology is shown in Figure 3.

The use of SC-CO_2_ miscible flooding is due to its excellent fluidity in the formation and its ability to expand the volume of crude oil when dissolved in it, thereby reducing its viscosity and lowering the interfacial tension of the crude oil [65]. In particular, heavy oil reservoirs with high viscosity and density have a good mutual solubility with SC-CO_2_, enabling a significant reduction in crude oil viscosity, which is very beneficial for the replacement of heavy oil [66]. At the same time, CO_2_ has the effect of a dissolving gas after dissolving in crude oil. CO_2_ separates from the crude oil and creates bubbles in the crude oil, pushing the crude oil to flow and improving the oil drive efficiency, when the formation pressure drops below the saturation pressure [67].

### 3.1. The Swelling of Heavy Oil by SC-CO_2_

The solubility of CO_2_ in crude oil is an important parameter for EOR and CCUS, while the volume expansion of crude oil is a mechanism for CO_2_-EOR. Consequently, it is necessary to investigate the mechanism of CO_2_ volume expansion under near-critical and supercritical conditions for enhanced recovery, which will provide us a better understanding of the chemical and physical reactions in the CO_2_-EOR process. The important mechanism of crude oil expansion involves CO_2_ easily dissolving into crude oil and causing it to expand, thus supplementing reservoir pressure and increasing oil saturation [68]. Volume expansion increases the internal kinetic energy of crude oil and reduces the capillary resistance and flow resistance in the process of crude oil flow, thus improving the flow capacity of crude oil [69]. The effect of expansion on heavy oil is mainly manifested in three aspects. The residual oil in the reservoir after water flooding is inversely proportional to the expansion coefficient, that is, the greater the expansion, the lesser the residual oil in the reservoir [70]. The dissolution of CO_2_ in oil droplets displaces water from the pore space, resulting in a process where the water-wet system becomes a drainage rather than water absorption. The relative permeability curve of oil discharge is higher than that of oil suction, creating an expedient flow environment under any given saturation condition [71]. On the one hand, volume expansion can significantly increase the oil saturation and formation elastic energy of the reservoir. On the other hand, the remaining oil after expansion can be separated or partially separated from the formation water and become mobile oil [72].

**Figure 4:**
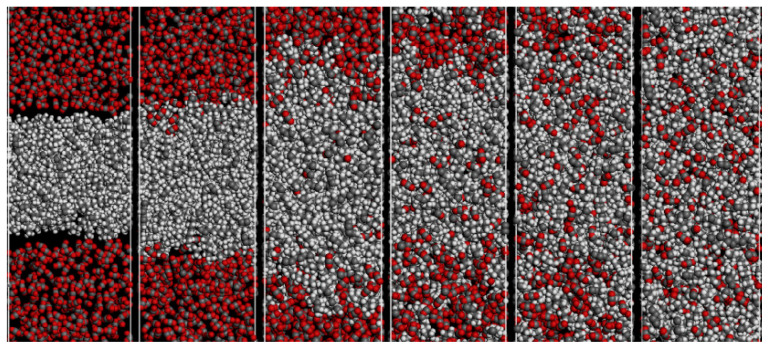
The microscopic process of the volume swelling for CO_2_–decane system. Color code: gray, carbon; red, oxygen; white, hydrogen [76].

Crude oil is a mixture composed of compounds such as n-alkanes, cycloalkanes, aromatic hydrocarbons, resins, and asphaltenes, making the interaction between CO_2_ and the constituents of crude oil exceedingly intricate. Since Whorton et al. applied for the patent of CO_2_ flooding in 1950, many experts and scholars have studied the phenomenon of volumetric expansion effect of dissolved CO_2_ in crude oil [73]. The alterations in the properties of crude oil are primarily as a result of CO_2_ dissolving in crude oil and are affected by the dispersion of CO_2_ in crude oil. Yang et al. analyzed the swelling behavior of oil by qualitatively discussing the dispersion properties of CO_2_ in the oil. The results show that the dispersion of CO_2_ molecules in the alkane phase plays a dominant role in the increase in the volume of CO_2_–alkane system. The solubility of CO_2_ in alkanes and the volume of the CO_2_–alkane system decrease with increasing alkane molecular length [74]. However, the expansion mechanism of CO_2_ dissolved in crude oil cannot be fully characterized under experimental conditions. In recent years, some researchers have investigated the interfacial properties and phase equilibria of the CO_2_–crude oil system by using molecular simulation techniques to understand the dynamic behavior of alkane solid surfaces. Zhang et al. investigated the solubility of CO_2_ in octane and its effect on the expansion of octane (n-octane) by performing configurational-bias Monte Carlo simulations in an osmotic ensemble. The results show that the interaction between octane and CO_2_ strengthens with increasing CO_2_ solubility in the pressure range of 2–10 MPa and at temperatures ranging from 323–353 K. The interaction between octane and CO_2_ is the main cause of octane dissolution [75]. Similar conclusions were reached by Liu et al., who found that the interaction between CO_2_ and decane molecules is the main reason for the volume swelling of the CO_2_–alkane system. The dispersive interaction between CO_2_ and n-decane molecules is the essence of the volume swelling of the CO_2_–n-decane system. At the same time, they noted that the volume swelling rate of decane was accelerated by the increase in the average spacing between decane molecules and the stretching of decane molecules. Figure 4 illustrates the microscopic process of volume expansion in the CO_2_–octane system [76].

**Figure 5:**
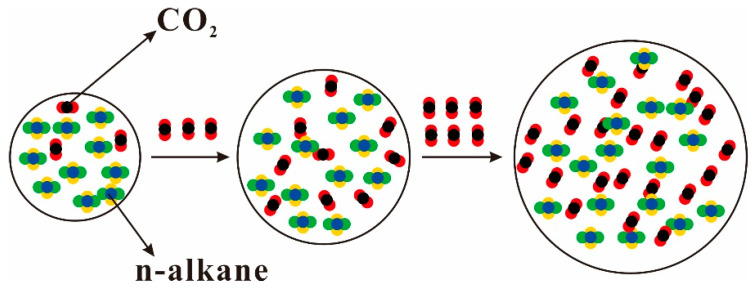
Diagram of volume expansion caused by CO_2_ dissolved in n-alkane (the mole fraction of CO_2_ is 25%, 50%, and 75%).

As the solubility of CO_2_ in oil increases, it leads to an increase in the distance between the oil molecules, and the force between oil molecules decreases, resulting in an expansion of the oil volume [77]. Figure 5 shows the process of dissolution and volume expansion of CO_2_ in n-alkane. As can be seen from the figure, when the molar fraction of CO_2_ is 25%, the molecules fill the space between the hydrocarbon molecules, forming a dispersed form. When the molar fraction reaches 50%, the overall volume of the system expands to a certain extent, and the CO_2_ and hydrocarbon molecules interpenetrate each other. When the CO_2_ mole fraction reaches 75%, the number of CO_2_ molecules is absolutely dominant, and the total volume of the system is greatly increased due to the dramatic increase in the number of both molecules. In addition, the longer the carbon chain and the larger the molecule of a hydrocarbon, the harder it is to dissolve CO_2_ [78].

**Figure 6:**
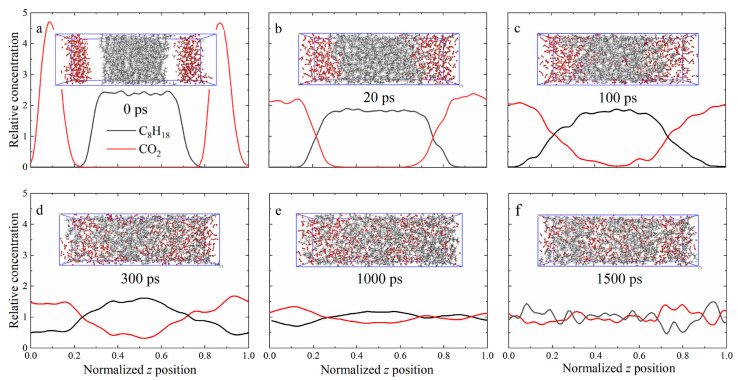
Simulation snapshot of swelling of CO_2_–octane system at P = 7.69 MPa and T = 333 K: (**a**) t = 0, (**b**) 20, (**c**) 100, (**d**) 300, (**e**) 1000, and (**f**) 1500 ps. Color code:C_8_H_18_ (black) and CO_2_ (red) [80].

Normal n-alkanes with shorter chains have smaller negative intermolecular interaction energies, which means that the molecules are more easily separated from each other, and therefore a higher swelling factor can be observed when octane is saturated by CO_2_. The interaction between alkane chains decreases with increasing pressure, indicating that the diffusion capacity of oil molecules increases when more CO_2_ molecules are dissolved by heavy oil [79]. In order to observe the CO_2_ dissolution process, Wang et al. observed the CO_2_–octane system by using molecular dynamics simulation methods. At the initial stage, some CO_2_ molecules begin to penetrate into the octane phase, resulting in a slight expansion of the octane volume. As a result, the CO_2_–octane system expands further, providing more space for the diffusion of CO_2_. The swelling process of the CO_2_–octane binary system is shown in Figure 6 [80]. It was also found that when CO_2_ was injected into the system, the alkane chain was slightly extended, meaning that the presence of CO_2_ molecules caused the alkane molecules to expand, which further led to the expansion of the systemic volume.

Regarding the study on the swelling behavior of CO_2_ with cycloalkanes, Sima et al. studied the vapor–liquid equilibrium of binary systems of CO_2_ with cycloalkanes (cyclopentane and cyclohexane) and showed that CO_2_ is more soluble and miscible in n-alkanes than in cycloalkanes [81]. Similar conclusions were obtained by Zhang et al. in their study of the solubility and volume expansion factors of CO_2_ in cycloalkanes. They believe that as the number of carbon atoms increases, the dispersibility between alkane molecules increase, the interactions between alkane molecules are enhanced, and the solubility of CO_2_ in alkanes decreases [82]. A large number of research works have investigated the solubility and volume expansion mechanism of CO_2_ in single alkane, brine, and alkane–water binary systems, while the microscopic mechanism of CO_2_ in hydrocarbon mixtures is not sufficiently clear. Therefore, the study of CO_2_ in hydrocarbon mixtures has received much attention. Wang et al. measured the volume expansion of CO_2_ and n-dodecane in fused silica capillaries using Raman spectroscopy. The results show that the expansion coefficient increases with the increase in pressure and decreases with the increase in temperature [83]. CO_2_ is less soluble in cycloalkanes and aromatics than in n-alkanes with the same number of carbon atoms. It is concluded that the solubility of CO_2_ in hydrocarbon mixture is mainly affected by high-carbon hydrocarbons through the above study, i.e., the higher the proportion of high-carbon hydrocarbons in the mixture, the lower the solubility of CO_2_ [84].

### 3.2. SC-CO_2_ Viscosity Reduction Characteristics in Heavy Oil

SC-CO_2_ is characterized by a large diffusion coefficient and a strong solvency capacity, and it can rapidly penetrate into the mixture [85,86]. When CO_2_ is dissolved in crude oil, the attraction force between oil molecules becomes smaller and the internal friction during flow is reduced due to the carboxylation reaction, thus the viscosity of the oil is reduced and the fluidity of the crude oil is improved. For heavy oil reservoirs, when a certain concentration of CO_2_ is injected into heavy oil, the internal intermolecular force changes from the liquid–liquid intermolecular force to the liquid–gas intermolecular force, and the intermolecular force is greatly reduced. Furthermore, the gum asphalt macromolecular structure of heavy oil will also be destroyed after dissolving CO_2_, the volume will be expanded, the density will be reduced, and the viscosity will be rapidly reduced [87].

**Figure 7:**
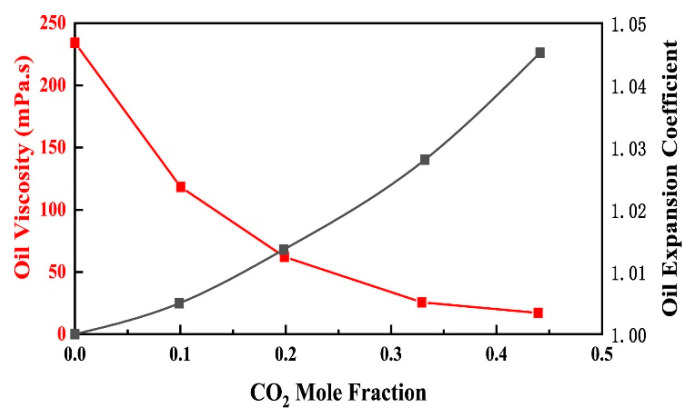
Effect of CO_2_ mole fraction on oil expansion coefficient and oil viscosity [91].

The high content of asphaltene and gum in heavy oil leads to adsorption of micelles on gum to form a solvation layer. Therefore, the dispersed phase occupy a large volume fraction, the attractive interaction between the particles of the dispersed phase is strong, and the internal friction is large, so it has a high viscosity. The stronger is the interaction between colloidal particles and solvent, the higher is the degree of solvation, the higher is the bound solvent, the greater is the viscosity of the system. In recent years, researchers have conducted a lot of research on the effect of CO_2_ flooding on viscosity reduction in heavy oil. Seyyedsar et al. suggested that CO_2_ can cause methane in heavy oil to be released in the form of microbubbles. The release of methane from heavy oil results in an increase in oil viscosity, and this increase is significant for heavy oil systems. However, the dissolution of CO_2_ in the oil will compensate for this viscosity increase. Experimental studies have shown that the saturation of CO_2_ in heavy oil is generally not linearly related to the viscosity reduction rate of heavy oil. In other words, as long as a small amount of CO_2_ is added to the oil, the viscosity of the heavy oil can be greatly reduced [88,89]. In order to further prove the viscosity reduction effect of CO_2_, Sun et al. compared the viscosity reduction effect of CO_2_ and nitrogen on heavy oil. The experimental results show that the viscosity reduction effect of CO_2_ on heavy oil is much better than that of N_2_ under the same conditions. Meanwhile, with the increase in CO_2_ pressure, the shear thinning feature becomes more and more obvious. With the decrease in temperature, the viscosity reduction rate of CO_2_ on heavy oil increases. The main mechanism is that when CO_2_ is dissolved in crude oil, the dispersion effect increases the distance between crude oil molecules, and ultimately reduces the viscosity of crude oil [90]. Regarding the effect of pressure on the viscosity reduction in CO_2_, Wang investigated the viscosity reduction in heavy oil by CO_2_ flooding through a combination of software simulation and experiment. The main findings are shown in Figure 7 below. With the increase in CO_2_ injection, the critical pressure of CO_2_–heavy oil system gradually increases, and the saturation pressure at reservoir temperature also gradually increases, and the viscosity of crude oil rapidly decreases as CO_2_ is injected [91]. We can conclude that the affinity of CO_2_ to asphaltene is greater than that of gum, and it can be adsorbed on asphaltene in the state of small molecules, replacing the original gum adsorption layer (solvated layer). On one hand, CO_2_ releases small molecules that are bound in the solvation layer, reducing the attraction between molecules, allowing them to move freely, resulting in a decrease in system viscosity. On the other hand, CO_2_ molecules are too small to form a thick adsorption layer, so that the asphaltene micelle are exposed. The volume fraction of the dispersed phase in the system is greatly reduced, and it is not easy for the micelles to interact with each other. The above factors cause a decrease in the viscosity of the heavy oil system.

The dissolution of CO_2_ can significantly reduce oil viscosity has been reported in the literature, especially for heavy oil. The solubility of CO_2_ in three heavy oils with different densities and the corresponding crude oil viscosity and density have been studied by Miller et al. at different temperatures and pressures. When there is no CO_2_, the viscosity of crude oil increases with the increase in pressure, while when there is CO_2_, the viscosity of crude oil decreases with the increase in pressure, and the viscosity of crude oil containing CO_2_ is significantly reduced with the increase in temperature [92]. Li et al. studied the effect of CO_2_ on the expansion and viscosity reduction in crude oil through experiments. The experimental results show that the viscosity of the dissolved CO_2_ containing oil decreases with the increase in saturation pressure. When the formation pressure is 23 MPa, the viscosity decreases by 44% [93]. Zhao et al. studied the effect of CO_2_ on the physical properties of high pour-point oil. The experimental results show that the solubility of CO_2_ in high pour-point oil decreases with the increase in temperature. At the same temperature, the viscosity of high pour-point oil decreases as the amount of dissolved CO_2_ increases, and the more dissolved CO_2_ there is, the greater the decrease in viscosity. The viscosity of pour-point oil is 27.0 mPa·s at the formation temperature of 55 °C, and the viscosity of crude oil fully saturated with CO_2_ decreases to 10.2 mPa·s, with a decrease of 62.2% [94]. Xin et al. measured the viscosity reducing effect of CO_2_ on crude oil in the North China NB reservoir under an experimental pressure of 15.9 MPa and a temperature of 94 °C. The experimental results show that the gas–oil ratio of the crude oil saturated with CO_2_ increased from 34 m^3^/t to 114 m^3^/t, and the viscosity of the crude oil decreased from 3.41 mPa·s to 1.92 mPa·s, a reduction of about 34% [95]. Zhao et al. carried out a feasibility study on CO_2_ flooding in ultra-low-permeability reservoirs using laboratory experiments and numerical simulation. Similar to the results of many of the above researchers, it is observed that the amount of dissolved CO_2_ in oil increases with the increase in pressure, and the viscosity of the heavy oil rapidly decreases. Under the formation pressure (18.18 MPa), the dissolved oil–gas ratio is 80.07 m^3^/t, and the crude oil viscosity is 1.745 mPa·s. When CO_2_ is injected under this pressure, the formation oil viscosity is 1.104 mPa·s, and the crude oil viscosity is reduced by 36.73% [96]. Liu et al. studied the viscosity reduction effect of CO_2_ flooding on ultra-low-permeability reservoir in Daqing Oilfield by using thin tubes and natural core. The experimental results show that under the formation pressure (18.2 MPa), the dissolved oil–gas ratio is 80.07 m^3^/t, and the crude oil viscosity is 1.745 mPa·s [97]. After the CO_2_ was injected, the oil–gas ratio is increased to 236.06 m^3^/t, and the viscosity of formation oil is 1.104 mPa·s, the viscosity of crude oil is reduced by 36.73%, and the fluidity of crude oil is increased. CO_2_ dissolved in crude oil significantly reduces the viscosity of crude oil, and the higher the viscosity of crude oil, the greater is the reduction [98]. From the above data, we conclude that CO_2_ has high solubility in heavy oil, and the complete miscibility of CO_2_ with heavy oil can dilute heavy oil, which is the main reason that the viscosity of heavy oil decreases greatly and the recovery efficiency increases.

### 3.3. SC-CO_2_ Extraction

The principle of SC-CO_2_ extraction and separation is to use pressure and temperature to control the dissolution capacity of supercritical fluid, and then achieve the extraction and separation of substances. Hydrocarbons and various lipid organic compounds in the reservoir medium can be extracted when CO_2_ reaches supercritical state, and the material inside the matrix are dissolved in the process and flow out of the pores with the gas [99,100]. The pore is widened and the crude oil adsorbed on the matrix is resolved due to the extraction of SC-CO_2_, which changes the heavy oil from the adsorbed state to the free state, thereby causing the crude oil to flow out of the pore channels. This process also expands the passage of crude oil, allowing gas to enter the matrix more easily and also allows the crude oil to be extracted quickly.

Kesavan et al. investigated the recovery of oil from oil shale using supercritical extraction technology with CO_2_ as the solvent. Their work successfully demonstrated the feasibility of using CO_2_ as a solvent for extracting oil from oil shale, completing groundbreaking research on the utilization of SC-CO_2_ for oil shale extraction [101]. Deo et al. found that two solvents, CO_2_ and propane, exhibited strong extraction capacity for crude oils from light-chain alkanes to heavy-chain alkanes around their respective critical temperatures [102]. With further studies on crude oil extraction, Guiliano et al. found that only n-alkanes between C_14_ and C_24_ were present in the extract solution at a pressure of 130 bar, with few branched chain alkanes and cycloparaffins present. Normal alkanes were extracted up to C32 at a pressure of 300 bar. The results showed that with the increase in pressure (130–300 bar), a greater variety of branched chain alkanes and cycloparaffins were extracted, along with heavier aromatic compounds [103]. A similar study was performed by Mihkel Koel et al., which was different from Guiliano as they studied the application results of SC-CO_2_ extraction at different temperatures. They found that pure CO_2_ preferentially extracted n-alkanes [104]. Rudolf et al. studied the extraction of aliphatic and aromatic hydrocarbons from shale rock using SC-CO_2_. SC-CO_2_ was found to be effective in extracting “free” hydrocarbons from samples in a lower temperature range [105].

**Figure 8:**
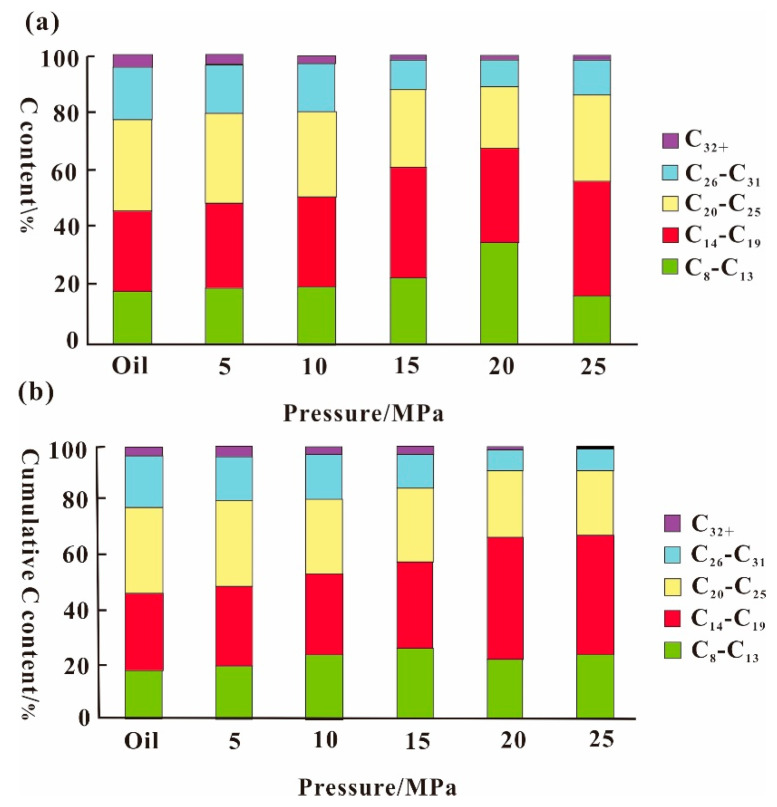
(**a**) Carbon component under continuously ascending pressure. (**b**) Carbon component under individual pressure after CO_2_ breakthrough [107].

**Figure 9:**
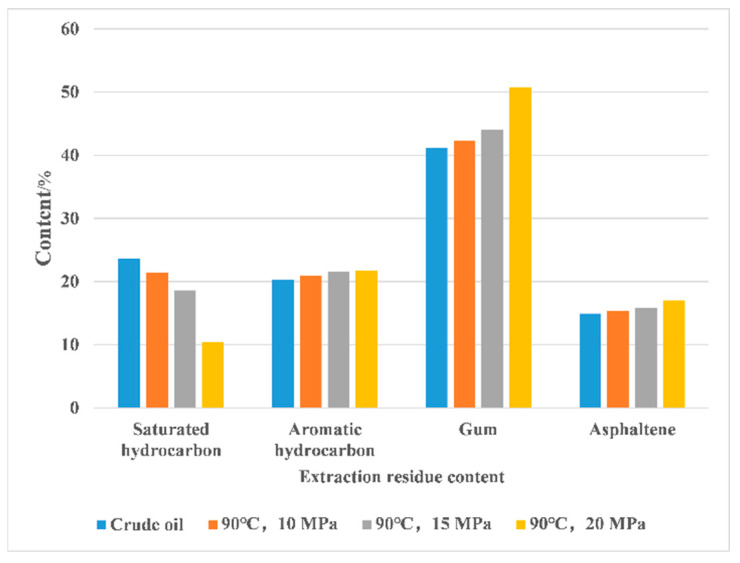
The percentage of extraction residue component [109].

Studies have shown that SC-CO_2_ mainly extracts components below C_20_, and the existence of a pressure critical point in this extraction process ensures that the extraction proceeds smoothly [106]. As can be seen in Figure 8a, the carbon fraction of the oil production remains essentially unchanged before the gas breakthrough. From Figure 8a,b, it can be observed that at the same pressure of 25 MPa, the fractions of components below C_20_ are 56.3% and 65.3%, respectively, while the ratio of components below C_20_ in crude oil is 46.3% [107]. The extraction capacity of CO_2_ increases with the increase in its density. The appropriate increase in pressure increases the density of CO_2_, and the extraction is easier to carry out. Factors affecting the optimum extraction temperature include the increase in diffusion coefficient due to warming and the decrease in CO_2_ density, which together influence the magnitude of the optimum extraction temperature [108]. Peng et al. conducted a study on the content and viscosity of the components in the extracted material and concluded that the extraction of light hydrocarbon components from heavy oils is enhanced with increasing extraction pressure and temperature in CO_2_ extraction process. As can be seen from Figure 9, with the increase in extraction pressure, the percentage of light components in the residue after CO_2_ extraction is reduced by about 56% compared with the original oil sample. The results show that with the increase in pressure, the extraction effect of CO_2_ on super heavy oil is enhanced, and the light components are continuously extracted [109]. When the pressure reaches a high level, according to the colloidal system theory of heavy oil, the original stable colloidal structure of heavy oil is disrupted, and the heavy asphaltenes agglomerate, leading to asphaltene precipitation [110].

Allawzi et al. concluded that SC-CO_2_ extraction of oil is selective and preferentially extracts non-polar substances. It was found that CO_2_ mainly extracted saturated hydrocarbon, olefins, and some aromatics in crude oil [111]. Small aromatic hydrocarbons (such as methylbenzene, methyl naphthalene, etc.) exist in the form of micelles in crude oil, while paraffin exists in the form of colloids. The original equilibrium state of small saturated hydrocarbons and aromatic hydrocarbons is disrupted by the extraction of SC-CO_2_, leading to the deposition of macromolecular compound [112]. Ni et al. further studied the selective extraction of SC-CO_2_. It was found that SC-CO_2_ cannot completely extract all components from the oil, and it preferentially extracts saturates and leaves behind asphaltene. The results show that low-boiling and non-polar or low-polar petroleum components can be quickly extracted from rocks using SC-CO_2_ [113]. The selectivity of SC-CO_2_ extraction of crude oil molecules under different pressures and temperatures was studied by Cao et al. [114]. SC-CO_2_ preferentially extracts small molecules with relatively low polarity, while large molecules with relatively high aromaticity and polarity are not easily extracted using SC-CO_2_, and compounds containing multiple heteroatoms are more difficult to be extracted than hydrocarbons [115]. This is because non-polar saturated hydrocarbon molecules are less affected by the adsorption of other components, so they are easily extracted using SC-CO_2_. Aromatic hydrocarbons are less affected by the circumflex network structure of asphaltene and gum due to their smaller molecular size. In addition, SC-CO_2_ molecules have weak polarity, making it easier to dissolve aromatic hydrocarbon molecules and enhance the diffusion coefficient of aromatic hydrocarbons [116].

## 4. Research Progress on the Mechanism of CO_2_ Flooding Improved by Typical Oil Flooding Agents

### 4.1. Nanoparticles Inhibit Asphaltene Deposition during CO_2_ Flooding

**Figure 10:**
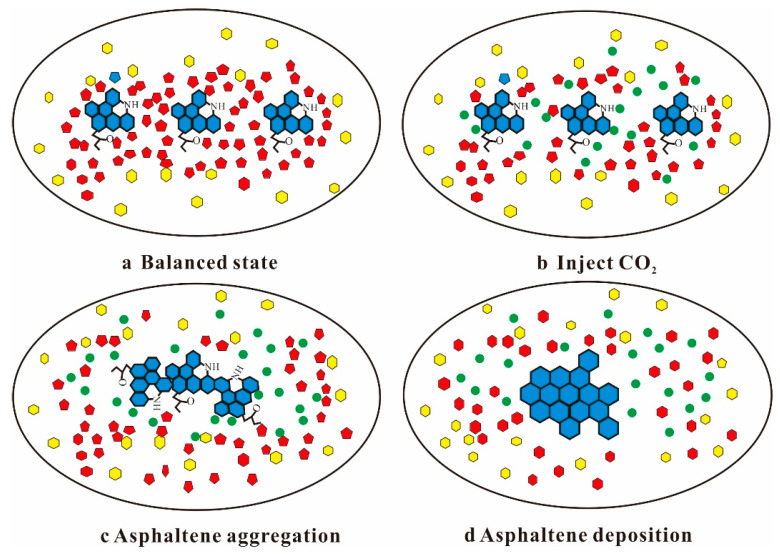
Asphaltene deposition during CO_2_ flooding, asphaltene (blue), aromatic hydrocarbon (yellow), gum (red), CO_2_ (green).

When CO_2_ gas is injected into the reservoir for oil displacement, the temperature and pressure in the reservoir change after CO_2_ molecules contact with crude oil, breaking the dynamic stability of the dispersion system. As shown in Figure 10, CO_2_ molecules compete with gum that coats the asphaltene surface for spatial positions after entering the crude oil. As a result, the force of forming the gum solvation layer is weakened, causing the gums that stabilize the asphaltene to continuously detach from the surface of the asphaltene particles [117]. Asphaltene particles that have lost their solvation layer coating may have attractive collision and aggregation under the interaction of electrostatic, dipole, and hydrogen bonding, which leads to asphaltene deposition [118]. The surface activity of asphaltene molecules is caused by the existence of polar groups, which will lead to self-association between asphaltene molecules, causing asphaltene aggregation and even deposition. This results in many problems such as blockage of reservoir pore throat, wettability reversal, and permeability decrease [119]. Therefore, solving or mitigating the issue of asphaltene deposition during CO_2_ flooding processes is crucial for enhancing oil recovery in low-permeability reservoirs.

#### 4.1.1. Adsorption of Nanoparticles

**Figure 11:**
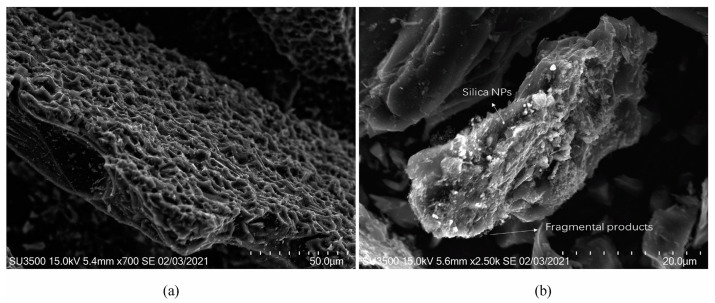
SEM image after carbonization at 800 °C: (**a**) oil–water emulsion and (**b**) oil–nanofluid emulsion [121].

Nanoparticles are considered to be one of the most promising solutions for asphaltene deposition due to their large specific surface area and adsorption capacity, better suspension and catalytic performance, and less susceptibility to reservoir damage compared to chemical agents. The strong polarity of the surface of asphaltenes is the fundamental reason for self-association and even deposition between asphaltene molecules. In the presence of nanoparticles, the strong polarity of asphaltenes can be exploited to inhibit asphaltene deposition and stabilize their dispersion in the crude oil system. By modifying the nanoparticles, on the one hand, the charge distribution on the surface of the particles can be changed, so that the particles will adsorb asphaltene after entering the reservoir, and asphaltene aggregation and deposition can be avoided. On the other hand, nanoparticles can be surface grafted so that the surface of the particles carries the corresponding polar groups, which can interact with asphaltene molecules after entering the reservoir and promote the stable dispersion of asphaltene. According to the DLVO theory, the strong polarity of the asphaltene and the surface charge of the nanoparticles facilitate an interaction between the two [120]. For example, silica nanoparticles containing hydroxyl groups and electronegativity can adsorb with heavy components that are interfacial activity. Moreover, silica nanoparticles can destroy the self-assembled structure of the heavy components of crude oil [36]. From Figure 11, it can be seen that silica nanoparticles are adsorbed on the collapsed layer, which proves that there is an interaction between silica nanoparticles and heavy components [121].

**Figure 12:**
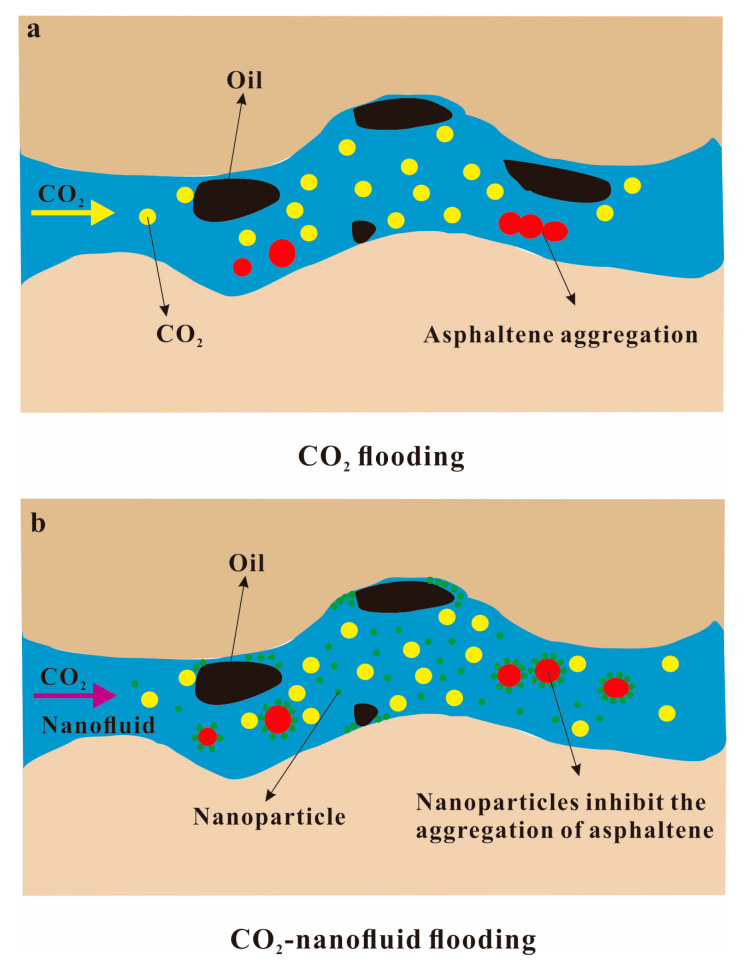
Adsorption inhibition of asphaltene deposition by nanoparticles: (**a**) CO_2_ flooding and (**b**) CO_2_–nanofluid flooding.

The surface charge of the nanoparticles can be adjusted by changing the pH of the nanofluid. Moreover, nanoparticles have strong fluidity in porous media and possess high surface activity and specific surface area. Therefore, they can adsorb the asphaltenes in crude oil, thereby inhibiting asphaltene deposition [122,123]. A Fe_3_O_4_–shell polyose nanocomposite was prepared by Panahi et al. This composite material exhibits a stronger capacity for adsorbing asphaltenes compared to single Fe_3_O_4_ nanoparticles. Its asphaltene adsorption isotherm is consistent with the Freundlich model, which further confirms the existence of self-association and multilayer adsorption of asphaltene on the nanoparticles [124]. Asphaltenes in crude oil tend to rapidly aggregate and deposit in low-permeability reservoirs during CO_2_ flooding. When the nanofluid is injected, the asphaltene will be adsorbed by the nanoparticles, making it stably dispersed in the crude oil system, as shown in Figure 12.

#### 4.1.2. Dispersion of Nanoparticles

**Figure 13:**
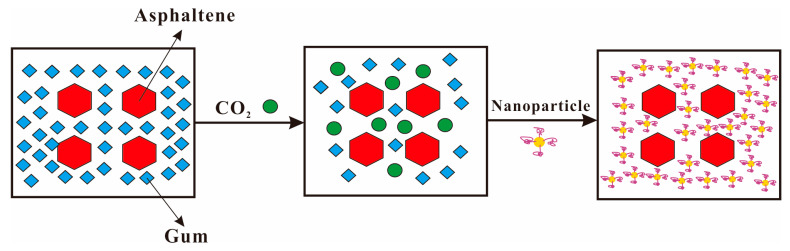
Schematic diagram illustrating the mechanism of nanoparticle dispersion inhibiting asphaltene deposition.

The self-association between asphaltene molecules will accelerate the flocculation and deposition of asphaltene. The dispersion of asphaltene by nanoparticles is mainly based on the formation of stable interaction force or space steric hindrance between the nanoparticles and asphaltene molecules. The dispersion effect disrupts the self-aggregation of asphaltene molecules, thereby inhibiting asphaltene deposition and removing asphaltene aggregates [125]. The surface-modified nanoparticles are prepared into nanofluid suspension and injected into the reservoir. When the nanoparticles enter the space between the gum and asphaltene, they can bind to asphaltene through dipole interaction, charge-transfer complexing interaction, and hydrogen-bonding interaction. An alkyl space stabilizing layer is established on the surface of asphaltene molecules, thereby dispersing the asphaltene and preventing its self-aggregation, and thus stabilizing the asphaltene and preventing it from being deposited [126]. As shown in Figure 13, the surface-modified nanoparticles are prepared into a nanofluid suspension and injected into the reservoir. When the nanoparticles enter the space between the gum and asphaltene, they bind together with the asphaltene through the effects of dipole interactions, charge transfer, and hydrogen bonding. The asphaltene molecules form a stable layer on the surface, which in turn disperses the asphaltene, avoiding self-aggregation and stabilizing the asphaltene to prevent deposition. Setoodeh found that the presence of multiple sulfur heterocycles in the structure of PT, which produce higher polarity in PT and can form stronger interactions with heteroatoms such as N, S, and O in asphaltene molecules. This creates more effective PT-coated nanoparticles than Fe_3_O_4_ nanoparticles in inhibiting asphaltene deposition [127].

Nanofluids can further strip the heavy oil in the pores where CO_2_ does not enter through the CO_2_ displacement channel. Nanofluids can change the wettability of the pore surface, so that the oil on the surface of the micropore is stripped into oil droplets during the CO_2_ flooding process. The advantage of the synergistic effect of the two is that nanofluids can not only change the reservoir wettability, but also increase the water injection capacity. Moreover, it can inhibit the pointing phenomenon and provide a larger sweep area for subsequent CO_2_ flooding [128,129]. In fact, nanoparticles are considered to be effective additives for increasing sweep efficiency, and can also increase the viscosity and density of SC-CO_2_. In Cao et al.’s experiment on water-based nanofluid-alternating-CO_2_ injection, it was found that the mass content of C10~C16 intermediate components in the crude oil extracted using NWAG was 9.55%, which was much lower than that of water-alternating-gas (WAG). The results show that after emulsification with nanofluids, CO_2_ can extract more intermediate components from crude oil, which improves the interaction between crude oil and CO_2_ [121]. The above studies focused on the development of mathematical models for water-based nanofluids, so SC-CO_2_-based nanofluids were studied by Dezfuli et al. The results indicate that for heavy oil reservoirs, the maximum ultimate recovery of heavy oil obtained by injection of SC-CO_2_ is 27.82% under the condition of 5.0 vol. % nano-particle concentration [130].

### 4.2. Polymer Enhanced CO_2_ Flooding

#### 4.2.1. Polymer Improves CO_2_ Channeling

CO_2_ flooding has the disadvantage of low sweep efficiency, which is mainly caused by gas flow in the high-permeability area of heterogeneous reservoir, viscous fingering caused by viscosity difference between oil and gas, and gravity override caused by density difference between oil and gas [131]. The main reason for CO_2_ gas channeling is that the viscosity of CO_2_ is much smaller than that of formation water and crude oil, and the viscosity pointing caused by the unfavorable mobility ratio, which makes the injected CO_2_ bypass the crude oil and crossflow occurs, and the sweep efficiency decreases. The heterogeneity of the reservoir causes injected CO_2_ to form channeling paths along high-permeability zones, which will affect the sweep efficiency [132].

**Figure 14:**
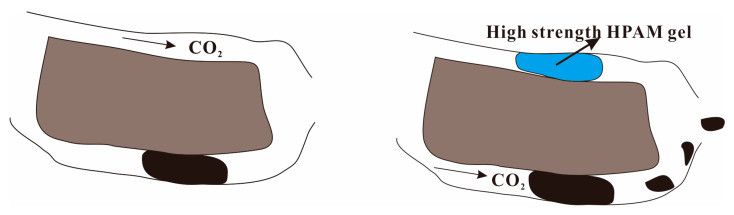
Schematic diagram illustrating channeling blocking with high-strength HPAM gel.

PAM gel is a conventional crosslinked gel polymer, which is mainly composed of polyacrylamide macromolecules and a crosslinking agent. It has the advantages of low adsorption loss, insensitivity to bacterial attack, and easy dissolution [133]. As a weak gel, HPAM preferentially enters the high-permeability region through porous media by flowing and undergoing stretching deformation along the large pores. This behavior can reduce the permeability of high-permeability layers, improve the intralayer heterogeneity of reservoir, expand the swept volume, and thus enhance oil recovery [134]. HPAM contains a large number of hydrophilic groups such as carboxyl and amide groups, and the electrostatic repulsion between carboxyl groups can unfold its main chain. The hydration and electrostatic repulsion of hydrophilic groups increase the volume of gel particles, showing a larger hydrodynamics radius. Simultaneously, the three-dimensional network structure formed by crosslinking allows it to possess a certain degree of strength, enabling it to create blockages in the deeper layers of the reservoir and alter the flow direction of the fluid [135,136]. As shown in Figure 14, Al-Ali et al. found that low concentration of HPAM gel has weaker strength and would flow with the produced oil, and only a small part played the role of blocking CO_2_. However, it is effective to inject high concentration HPAM gel to block the permeation channel or crack [137]. The study conducted by Sun et al. revealed that the polyacrylamide-chromium(III) polymer gel would be degraded when it is in contact with CO_2_. Therefore, after the gel in the long-term CO_2_ environment is degraded, a channel will be formed inside it, that is, the gel breaks through, thereby reducing the plugging efficiency of the polymer gel [138]. In summary, delayed crosslinked PAM can effectively block channeling and improve sweep efficiency, which is suitable for high temperature and high salt reservoirs. However, it has disadvantages such as uncontrollable gel time, easy blockage, and damage to the formation.

Preformed particle gel (PPG) is typically formed by PAM or HPAM with crosslinking agents on the ground to form gel particles. Both retarding crosslinking HPAM and pre-crosslinking gel particles diverting CO_2_ to the low-permeability zone by plugging high-permeability zones to improve swept efficiency. Zhou et al. found that AR-PPG exhibited better swelling and shear resistance in acidic environments. This is mainly due to the fact that the electrostatic repulsion between the cationic group and H+ and the steric hindrance effect between the sulfonic acid groups make the polymer chain difficult to be compressed, thereby increasing the acid resistance and shear resistance of AR-PPG [139]. The large presence of CO_2_ leads to an acidic environment in the reservoir during CO_2_ flooding. The development of PPG gel has resolved the issue of reduced plugging efficiency caused by the degradation of PAM in acidic reservoirs. IPN-ASSAP polymer gels were synthesized by Pu et al., which exhibit excellent CO_2_ responsiveness. CO_2_ will change the particle size, strength, and other properties of the particles, thereby improving the plugging capacity [140]. In conclusion, the research on PPG polymer gels has addressed the shortcomings of traditional PAM gels, which cannot remain stable in acidic environments for a long time. However, preformed particle gel still have the disadvantages of being too large in size, having a fast water absorption rate, and being difficult to inject.

#### 4.2.2. Polymer Enhanced Stability of CO_2_ Foam

In the subsurface environment, SC-CO_2_ has lower viscosity and the density of SC-CO_2_ is lower than that of water and heavy oil, which leads to the phenomenon of viscous fingering and gravity override in the process of reservoir seepage. The above phenomenon will lead to a reduction in its wave coefficient and seriously weaken the effect of the recovery rate. It has been demonstrated that CO_2_ foam can effectively adjust the section plane of the displacement front and reduce the fluidity of CO_2_ [141]. The generating ability and stability of foam can effectively improve the sweep coefficient of foam flooding. However, the high solubility of CO_2_ in water accelerates the diffusion rate of CO_2_ through the liquid film, resulting in CO_2_ foam generally being less stable than nitrogen foam [142,143]. Therefore, how to effectively improve the stability of CO_2_ foam has attracted much attention in recent years [144].

Polymer-reinforced foam is a kind of oil displacement system in which a polymer is added to the foam as a foam stabilizer. In recent years, polymer-reinforced foam systems have been extensively reported in CO_2_ foam flooding. Lv et al. found that with the addition of HPAM, the drainage rate of CO_2_ foam film decreased. The liquid inside the foam film is not easily lost, and the permeability of the liquid film is reduced, inhibiting the diffusion of gas within the foam. In the experimental range, when the concentration of HPAM is 0.025%, the CO_2_ foam half-life more than doubled and reached a maximum. If the polymer is added in excess, the foamability will be greatly reduced [145]. This is mainly because foam stability is primarily related to film drainage rate, Marangoni repair effect for the film, foam disproportionation rate, and liquid-sheet disintegration rate. The viscosity of the solution increases substantially with the addition of polymer. According to the film drainage rate formula, the film drainage rate decreases and the liquid film thickness increases when the solution viscosity increases. It also reduces the gas diffusion rate between the bubbles and decreases the foam disproportionation rate. On a macroscopic level, the foam exhibits higher stability [146].

$$V_{RE}=-\frac{dh_{f}}{dt}=\frac{2h_{f}^{3}}{3\eta_{c}r_{film}}\Delta P_{film}$$

$V_{RE}$: film drainage rate, m/s, $h_{f}$: liquid film thickness, m, *t*: time, s, $r_{film}$: liquid film curvature radius, m, $\eta_{c}$: solution viscosity, Pa·s, and $P_{film}$: pressure difference on both sides of liquid film, Pa.

Within a certain mass concentration range, polymers can increase the viscosity of the foam system, increase the thickness of the foam film, reduce the film drainage rate and diffusion rate of the foam, and enhance the stability of the foam [147]. This view was also confirmed in the experiments of Zhang et al. Hydroxypropyl methyl cellulose (HPMC) can be used as a foam stabilizer to effectively improve the stability of the foam, forming a polymer-reinforced CO_2_ foam system. However, the foaming ability of the foaming agent system will be weakened by excessively high polymer quality fraction [148]. In addition, the addition of PAM to the foam solution also improves the strength of the liquid film surface, thereby reducing the emission of liquid in the foam and minimizing the diffusion of the gas phase [149].

**Figure 15:**
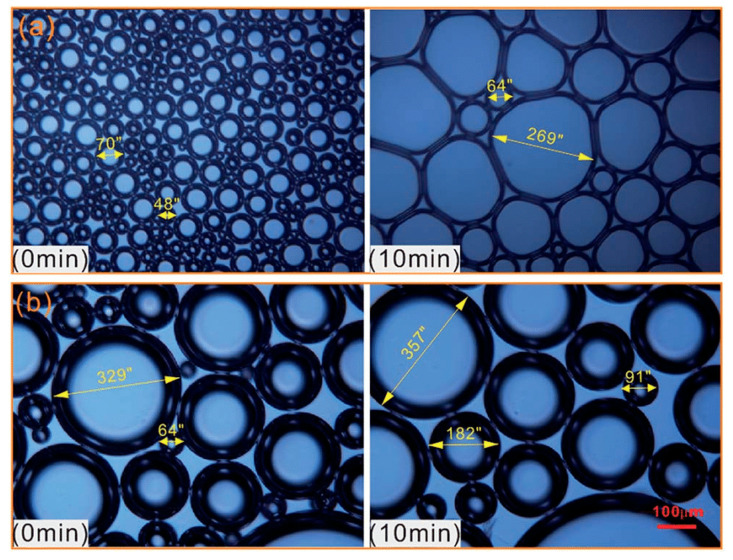
CHSB foam: (**a**) polymer-reinforced foam and (**b**) measurement of the size of foam over time [150].

**Figure 16:**
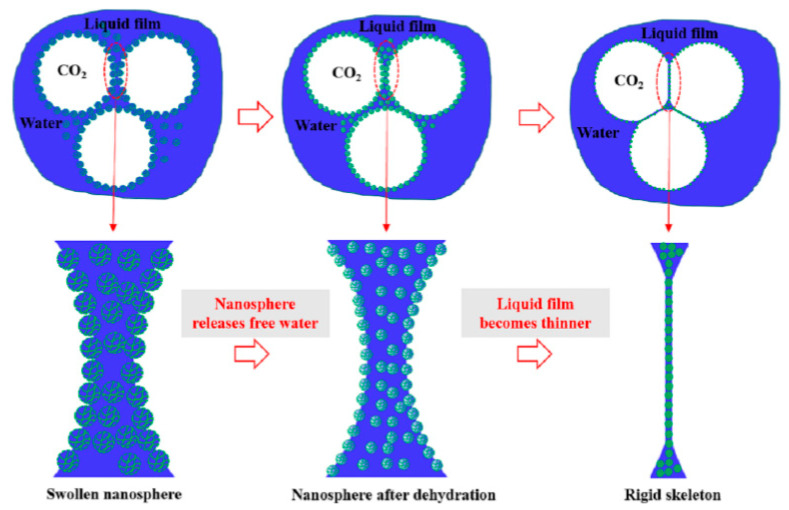
Mechanism diagram of polymer nanospheres stabilizing CO_2_ foam [41].

The above studies only introduce the stability mechanism of polymer on CO_2_ foam from the macro aspect, and some researchers have carried out relevant discussions on the micro scale. Polymer-reinforced properties of CO_2_ foam depend on the type of polymer. Pu et al. found that the CHSB-nonionic polyacrylamide system was more stable than the CHSB-anionic polyacrylamide system. Specifically, the foam reinforced with xanthan gum exhibited the best stability. As can be seen from Figure 15, the shape of the CHSB foam changes to a polyhedral shape over time, while the polymer-reinforced foam has a smaller change in size [150]. Yang et al. found that the three-dimensional network spatial structure of acid-resistant hydrophobic polymer nanospheres prompts them to form a “skeleton” at the gas–liquid interface. It increases the strength of the liquid film and regulates the viscoelasticity and liquid storage capacity of the liquid film. Polymer nanospheres swell and release free water, which prevents the rupture of liquid films and increases foam stability. Figure 16 shows the mechanism of stabilizing CO_2_ foam with nanospheres [41].

### 4.3. Surfactants Improve CO_2_ Flooding

In order to improve the effect of heavy oil mining, various additives are often used to assist CO_2_ to further increase the EOR. After the addition of a surfactant, the solubility of CO_2_ in crude oil under high pressure conditions increases, but the increase is not significant. The main reason is that CO_2_ is a non-polar substance, while surfactants have the capability to alter the properties of CO_2_, thereby increasing its solubility in crude oil [151,152]. In general, the carbon atoms on the carbon chain of surfactants (C_12_–C_18_) belong to the light components of crude oil. When the surfactant is added, it disrupts the initial oil–gas phase equilibrium of crude oil, so that CO_2_ can extract more light components in the crude oil [153]. The high content of macromolecular gums and asphaltenes in heavy oil is the main factor leading to a high value of the MMP. With the addition of surfactants, the internal structure of the molecules of the heavy oil is disrupted and their content is reduced, thus allowing the properties of the crude oil to change. Surfactants with polar groups such as ester groups and hydroxyl groups have the ability to form strong hydrogen bonds with hydroxyl or amino groups in gums and asphaltenes. Hydrogen bonding causes the molecular structure to be branched and the cohesive energy of the crude oil to be reduced, which plays a role in viscosity reduction [154,155]. The surfactant forms reverse micelle or microemulsion structure with CO_2_ and crude oil. The polar groups of surfactants form a polar core with the polar components in crude oil through hydrogen bonding. The polar groups of surfactants form a polar core with the polar components in crude oil through hydrogen bonding, and polar macromolecules such as asphaltenes and gums are encapsulated within it. The highly branched hydrophobic alkyl chains extend into CO_2_ and aggregate with each other by van der Waals force and thus increasing the extent of CO_2_ miscibility in crude oil [156,157,158]. When the surfactant spontaneously accumulates at the two-phase interface, the interaction between the CO_2_-philic end and CO_2_ is close to the interaction between the oil-wet end and crude oil. The pressure difference between the two phases decreases, causing the interfacial tension between the oil and gas phases to decrease.

#### 4.3.1. Surfactants on Foaming Properties and Foam Stability of CO_2_ Foam

The surfactant is injected before the CO_2_ injection, and foam is generated when the surfactant comes into contact with the CO_2_. It can selectively block the high-permeability channeling paths, improve the heterogeneity of the formation, and expand the swept volume, which effectively delays the time of CO_2_ channeling and prolongs the contact time between CO_2_ and the remaining oil [159]. Foaming ability and foam stability are two important properties to consider in CO_2_ foam flooding. Surfactants, with their characteristics of fast foaming speed, high foam output, and high foaming ability at lower concentrations, are typical chemical agents used in assisting CO_2_ flooding. Therefore, the foaming ability of surfactants has been extensively studied by researchers. Jones et al. suggest that as the kinetic energy of surfactant molecules increases, they are more likely to adsorb at the gas-liquid interface. A greater adsorption amount indicates a reduction in surface tension, which benefits the foaming ability of EAB surfactants [160]. This is because the generation of bubbles is related to surface tension, as shown in the following equation.

$$W_{e}=\frac{G\mu_{S}R}{\gamma }$$

G: shearing stress, $\mu_{S}$:viscosity of continuous phase, R: bubble radius, γ: IFT [161]. However, the link between interfacial tension and foamability has been viewed differently by researchers. Some scholars believe that the diffusion rate of surfactants increases with the maximum rate of decrease in interfacial tension. As a result, the diffusion rate of foaming properties also increases [162]. Another group of researchers believed that the excessive adsorption of surfactants leads to the disappearance of the interfacial tension differential value, which may result in damage of foaming or the cessation of the Marangoni effect [163]. The different perspectives arise from the different methods of forming CO_2_ foam [164,165]. Foam stability is determined by the interaction of interfacial tension, elasticity, temperature, and pressure. For example, when the bubbles expand, the decrease in surfactant concentration at the expansion site causes a change in interfacial tension. At this time, the surfactant migrates to the surface of the thin film, compensating for the thinning of the film [166].

**Figure 17:**
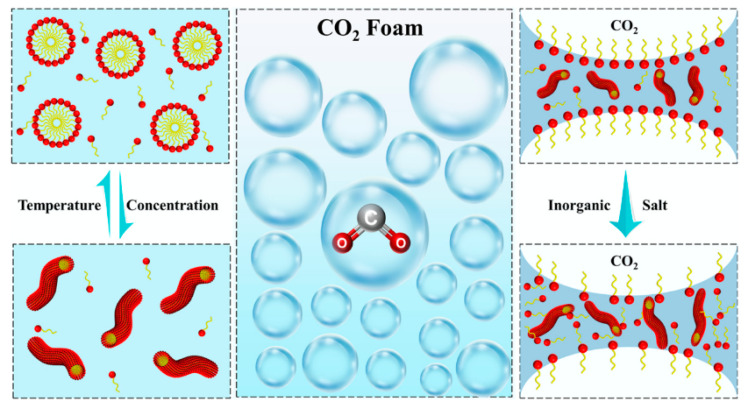
Schematic diagram of the CO_2_ foam stabilization mechanism and instability process for EAB [170].

CO_2_ dissolves in water, causing the aqueous solution to become acidic, which will accelerate the diffusion rate of CO_2_ through the liquid film. Therefore, it is difficult for conventional surfactants to maintain the stability of CO_2_ foam for a long time. Nonionic surfactant with an affinity for CO_2_ have attracted the attention of researchers [167,168,169]. Niu et al. discovered that EAB surfactants form worm-like micelles in foam solutions, increasing the diffusion resistance of CO_2_ between bubbles. At the same time, the worm-like micelles increase the interfacial viscosity and reduce the drainage rate of the liquid film. The mechanism of EAB surfactant stabilizing CO_2_ foam is shown in Figure 17 [170].

#### 4.3.2. Surfactants Reduce the Minimum Miscible Pressure (MMP) of CO_2_ Flooding

CO_2_ is a non-polar molecule, which has poor solubility with polar macromolecules such as gum and asphaltene in crude oil. In order to improve their miscibility, it is an effective method to add suitable surfactants to reduce the interfacial tension between CO_2_ and crude oil. Table 1 summarizes the experimental data of surfactants reducing the MMP between CO_2_ and crude oil.

CO_2_ is a weak solvent with low dielectric constant and low polarity, which causes most surfactants to be insoluble or slightly soluble in SC-CO_2_. The hydrophobic tail chain of the gas-soluble surfactants contain functional groups have an affinity for CO_2_, thereby enhancing the compatibility between surfactants and CO_2_. In addition to this, it has a low cohesive energy density, reducing the interaction between CO_2_ and surfactants. The branched chain on the hydrophilic group increases the space steric hindrance, and the more flexible the molecular chain is, the better the compatibility with CO_2_. Luo et al. showed that the surfactant C_i_PO_j_ significantly reduced the IFT between CO_2_ and crude oil, and the extent of CO_2_ miscibility with crude oil could be directly observed. In the scenario where the dosage of C_i_PO_j_ is 0.6% and the temperature is 60 °C, the MMP and first contact miscibility pressure of the CO_2_ and crude oil systems decreased from 19.1 MPa and 43.0 MPa to 13.8 MPa and 19.0 MPa, respectively [176]. The study by Sun et al. found that diisobutyl citrate can reduce the MMP between CO_2_ and oil. Injecting surfactants through the slug method can improve the EOR compared to CO_2_ flooding [181]. Currently, research on reducing the MMP between CO_2_ and oil systems using gas-soluble surfactants is in its preliminary stage, with shortcomings such as low degradability and high toxicity, which limits their application.

**Figure 18:**
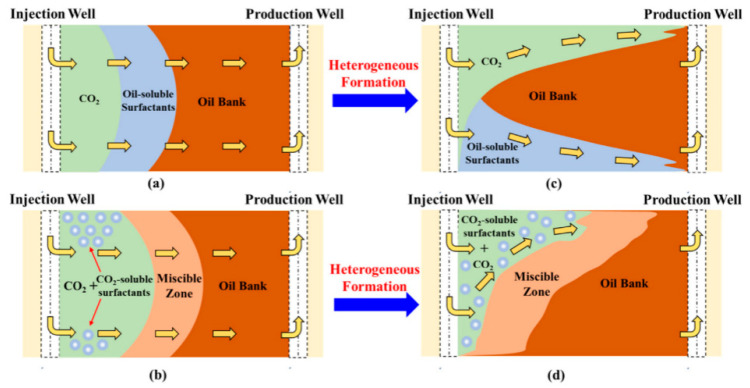
Reducing the MMP of CO_2_ and crude oil using (**a**) oil-soluble surfactants and (**b**) CO_2_-soluble surfactants, especially in the heterogeneous formation using (**c**) oil-soluble surfactants and (**d**) CO_2_-soluble surfactants [179].

Based on the above reasons, oil-soluble surfactants have attracted the attention of researchers. The study conducted by Wu et al. demonstrates that adding a concentration of 0.1% to 0.5% of surfactant (composed of a mixture of five surfactants) in crude oil can effectively reduce the oil–gas interfacial tension. Among them, the addition of 0.5% mix-benzene (aromatic hydrocarbon mixture) achieved the highest reduction, reaching 25.51%. The addition of mixed benzene increases the light hydrocarbon component of the crude oil and reduces the viscosity of the crude oil, which is conducive to the dissolution of CO_2_ in the crude oil and reduces the interfacial tension between the CO_2_ and the crude oil [171]. The oil displacement mechanism of oil-soluble surfactants and CO_2_-soluble surfactants are compared in Figure 18, highlighting the limitations of oil-soluble surfactants. The development of amphiphilic surfactants is the main research direction at present [179]. Yang et al. found that acetyl glucose is a CO_2_-philic end and aliphatic hydrocarbon is oil-wet end. Therefore, the amphiphilic acetyl glucose dodecyl ester can reduce the interfacial tension, and the addition of a 1% concentration of the reagent reduced the MMP by 27.47% [155].

## 5. Conclusions and Prospect

Low-permeability heavy oil reservoirs typically have low permeability and high porosity, which pose challenges to the flow of CO_2_ and its oil displacement efficiency. CO_2_ may encounter greater resistance in low-permeability reservoirs, resulting in poor mobility and limiting its diffusion capacity within the reservoir, leading to lower recovery. Heavy oil has higher viscosity and temperature, which increases the complexity of the interaction between CO_2_ and oil, and the solubility and diffusion rate of CO_2_ are influenced by temperature and viscosity. In addition, in low-permeability heavy oil reservoirs, it is a challenge for SC-CO_2_ to maintain the required temperature and pressure throughout the reservoir. Low-permeability heavy oil reservoir typically exhibit reservoir homogeneity, resulting in differences in rock properties and fluid distribution. This heterogeneity will affect the sweep efficiency and the distribution of CO_2_ in the reservoir, resulting in uneven recovery. Due to the low permeability of the reservoir, the transfer rate of CO_2_ is slow, and it is easy to take shortcuts, which will also lead to uneven distribution of CO_2_. Meanwhile, the migration path and dose control of CO_2_ are the key issues. In addition, CO_2_ can affect wellbore integrity, lead to cement degradation or casing corrosion, and can cause reservoir damage such as oil well plugging and pore collapse, thus impacting the long-term stability of the well. Ensuring the safe capture, transport, and storage of CO_2_ and reducing its impact on the environment is also a major challenge.

The article provides a detailed description of the main oil recovery mechanisms of SC-CO_2_ in low-permeability heavy oil reservoirs, as well as the improvement effect of nanoparticles, polymers, and surfactants on the defects of CO_2_ flooding. The dissolution of CO_2_ expands the volume of heavy oil, which can increase the elastic strain energy of formation, and thus increase the internal kinetic energy of crude oil. Capillary resistance and flow resistance are greatly reduced, the volume of formation void is increased, the flow of oil flow in rock pores is promoted, and the oil recovery rate is improved. CO_2_ interacts with heavy oil molecules through physical adsorption and chemical reactions. The infiltration of CO_2_ disrupts the internal structure and arrangement of heavy oil, breaking the adhesion force between oil molecules and reducing the viscosity of heavy oil, thereby improving its flowability. CO_2_ extracts light and heavy components in heavy oil or vaporizes them, thereby reducing the surface tension of oil–water interface, reducing residual oil saturation, and thereby increasing crude oil recovery. Asphaltene deposition, gas channeling, stability of CO_2_ foam, and reduction in minimum reverberation pressure are all problems to be considered in CO_2_ flooding. Therefore, this paper focuses on the improvement mechanism of nanoparticles, polymers, and surfactants on CO_2_ flooding. In response to the above challenges and the progress of current research, more laboratory studies and numerical simulations are needed to understand the interaction and flow mechanisms between CO_2_ and heavy oil. In addition, new dosage control methods need to be further developed to enhance the fluidity and solubility of CO_2_, and advanced monitoring and evaluation techniques should be employed to optimize the SC-CO_2_ flooding process.

## Figures and Tables

**Table 1:** Experimental data of surfactant reducing MMP of between CO_2_ and crude oil.

Surfactant	Gas	MMP Reduction/%	Method	Reference
Acetyl glucose dodecyl ester	CO_2_	27.47	IFT method	[155]
Multiple	CO_2_	17.86	IFT method	[171]
Oil-soluble	CO_2_	17.86	VIT	[172]
Surfactants	CO_2_	16.0–22.0	Observation cell	[173]
QDM, QHB	CO_2_	15.0	IFT method	[151]
Lauryl alcohol polyoxypropylene ether	CO_2_	25.6	-	[174]
Surfactant CAE	CO_2_	20.0	Slim-tube	[175]
Propoxylated	CO_2_	27.7	VIT	[176]
Oil-soluble	CO_2_	8.28	IFT method	[177]
C_1_, C_2_, C_3_	CO_2_	13.2	Slim-tube	[178]
NP-9	CO_2_	5.72	IFT method	[179]
2EH-PO_5_-EO_9_	CO_2_	8.03	IFT method	[179]
Amphiphilic	CO_2_	20.0	Slim-tube	[180]

## Data Availability

Not applicable.
